# Vision Deficit Due to Pituitary Apoplexy

**DOI:** 10.7759/cureus.38649

**Published:** 2023-05-06

**Authors:** Siddharth Shah, Rumana Khan, Keenan Bayrakdar, Christian Scott

**Affiliations:** 1 Internal Medicine, LewisGale Medical Center, Salem, USA

**Keywords:** ct and mri brain, secondary amenorrhea, serum prolactin, pituatary tumor, pituatary apoplexy

## Abstract

Pituitary apoplexy means “sudden death” of the pituitary gland, usually caused by hemorrhage or infarction and often occurring in a pre-existing pituitary adenoma. In many cases, pituitary apoplexy is a medical and surgical emergency. Fast, efficient diagnosis and treatment are important in many cases. This case exemplifies an ideal lab workup and referral process to turn out best outcomes and prevent medical complications in our patient.

## Introduction

Pituitary apoplexy is a rare and potentially life-threatening condition that usually occurs with co-existing pituitary tumor [1]. Headache is a common association, however, other symptoms such as decreased visual acuity, nausea, vomiting, confusion, ptosis, and diplopia are also seen [2]. Our patient is a young female who had gradual onset of symptoms. A complete endocrine workup done including MRI brain revealed a cystic lesion in the pituitary fossa compressing the optic chiasm. Surgery was performed resulting in immediate vision improvement.

## Case presentation

**Figure 1:**
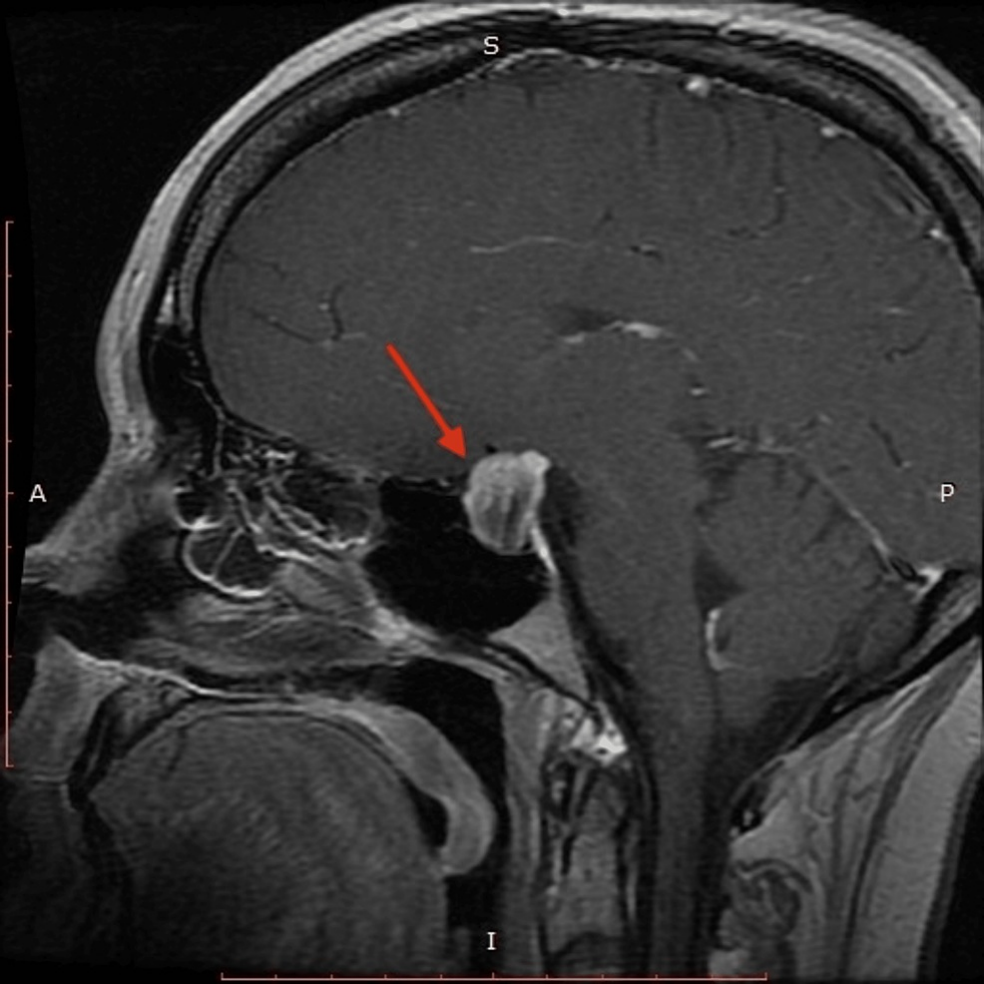
MRI of pituitary fossa compressing the optic chiasm

A 29-year-old female with a past medical history of generalized anxiety disorder and major depression presented to her primary care physician’s office for complaints of irregular menses for over three years. More recently, she was increasingly fatigued, had a 25-pound weight gain with heat intolerance and missed her menstrual cycles last four months. She visited her ophthalmologist during this time as she noticed changes in her vision, particularly a loss of her bilateral peripheral vision. She was told she had a left worse than right visual field deficit that was most notable in the upper outer quadrant. Ophthalmology subsequently referred her back to primary care physician for further lab work. A complete endocrine workup ensued including laboratory workup, imaging, and subsequent pituitary resection as shown in Table 1 and Figure 1.

**Table 1:** Normal lab values prior to surgery were not rechecked after the surgery LH: luteinizing hormone; FSH: follicle-stimulating hormone; TSH: thyroid stimulating hormone; ACTH: adrenocorticotropic hormone

Laboratory test	Before resection	After resection	Reference range
Prolactin	1258 ng/ml	26.2 ng/ml	4.8-23.3 ng/ml
LH	<0.3 mIU/ml	N/A	1.2-7.8 mIU/ml
FSH	1.6 mIU/ml	N/A	0.1-7 mIU/ml
TSH	1.3 uU/ml	N/A	0.5-4.7 uU/ml
ACTH	48.8 pg/ml	N/A	7.2-63.3 pg/ml
Total cortisol	67.5 ug/dl	N/A	3.4-22.5 ug/dl
24 hour urinary cortisol	13 mcg	N/A	6-42 mcg

**Figure 2:**
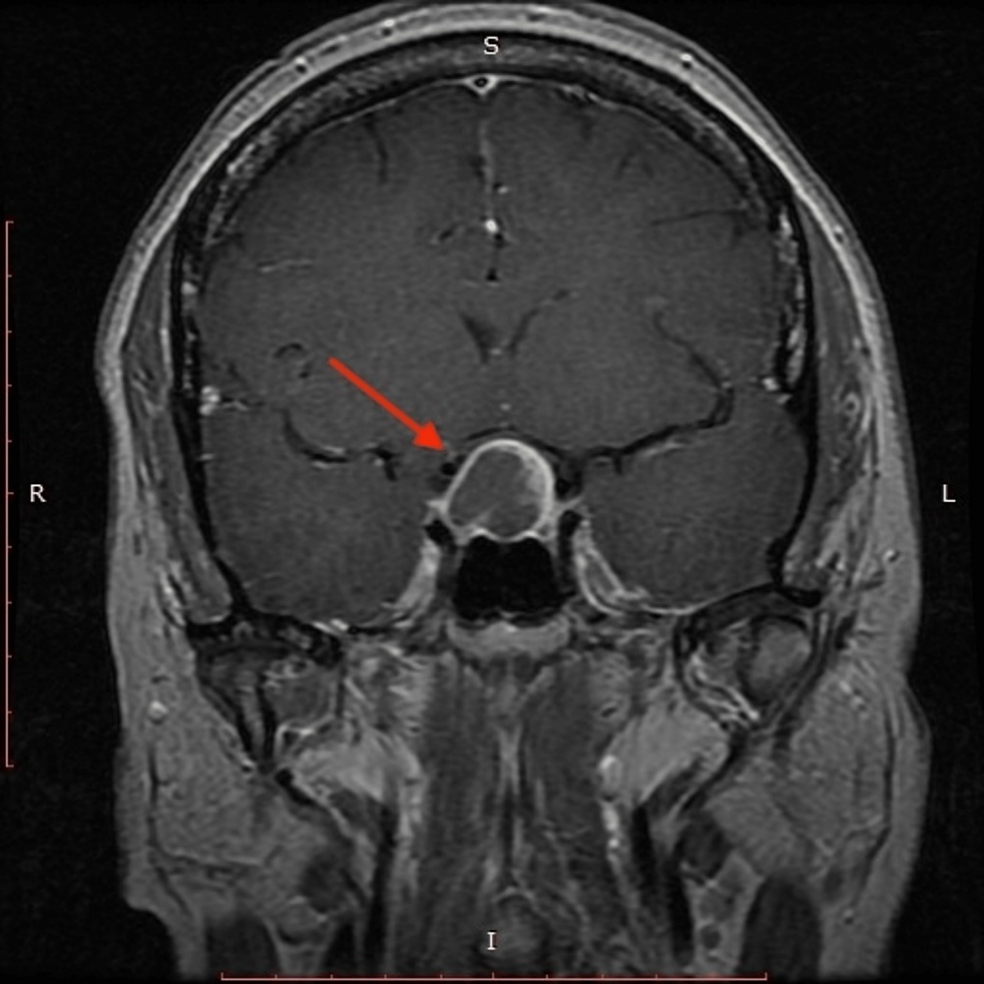
MRI of pituitary fossa compressing the optic chiasm

MRI of the brain demonstrated a 2.1x1.5x2.0 cm cystic lesion in the pituitary fossa compressing the optic chiasm. The radiographic differentials included a complicated Rathke cleft cyst, craniopharyngioma and or necrotic/cystic pituitary macroadenoma (Figure 2).

Given the subacute presentation of this patient with the radiographic appearance, pituitary macroadenoma with a subacute pituitary apoplexy was suspected. Given her progressively worsening presentation, the patient elected to proceed with trans-nasal transsphenoidal resection of the lesion from the sella and decompression of chronic-appearing blood and cystic fluid noted intraoperatively. An attempt made to find solid portions of the tumor was unsuccessful. Some normal gland was noted, but there was no evidence of a residual sella tumor. It was implied to have been destroyed during the apoplexy event. Surgery was completed successfully as evidenced by an immediate improvement in the patient’s post-operative vision exam.

Endocrinology was consulted and hydrocortisone was started for corticosteroid support of the hypothalamus-pituitary-adrenal (HPA) axis. This is standard of care to prevent comorbid secondary adrenal insufficiency. Electrolytes and urine output were monitored for possible central diabetes insipidus and to determine if further long-term therapy with desmopressin (DDAVP) would be needed. Sliding scale insulin was used for hyperglycemia secondary to steroid use during her admission. She was discharged home two days post-operation with a plan for close follow-up with the neurosurgeon and endocrinologist. 

At one week post-operative follow-up appointment for suture removal, the patient was doing well with significant improvement in vision.

## Discussion

Pituitary apoplexy is a rare emergency which can occur due to infarction or hemorrhage of the pituitary gland. The disorder most often involves a pituitary adenoma. Patients usually present with headache, vomiting, altered sensorium, visual defect and/or endocrine dysfunction [1]. Pituitary tumor apoplexy, although rare, carries a significant risk of morbidity and mortality. In the presence of a pituitary adenoma with apoplexy, neurosurgery is usually necessary. In some cases, post-surgical water balance disorders may be present due manipulation of the pituitary.

In our patient, the diagnosis of apoplexy and triphasic diabetes insipidus (DI) was based on unexplained hypotension in the setting of pituitary intervention. The patient later exhibited symptoms consistent with diabetes insipidus. Transient diabetes insipidus is not a common feature of pituitary apoplexy and is reported in around 2-20% of cases [2]. The hormones that are affected after a pituitary injury are growth hormone (GH) (88%), adrenocorticotropic hormone (ACTH) (66%), thyroid stimulating hormone (TSH) (42%), follicle-stimulating hormone (FSH) and luteinizing hormone (LH) (85%) [1,3]. In practice, LH and FSH are the first to be affected, while thyroid hormones are the last to be affected.

Triphasic DI is characterized by an initial polyuric phase. This can last up to four to five days and can lead to hypernatremia if the patient loses too much free water because of increased urinary free water clearance. In the second phase, transient syndrome of inappropriate antidiuretic hormone secretion (SIADH) occurs as a result of leakage of vasopressin from the damaged posterior pituitary tissue. This occurs five to six days post pituitary insult and can lead to hyponatremia. This hyponatremia can be exacerbated by the administration of free water during the initial hypernatremic phase [4,5]. 

The third phase, chronic diabetes insipidus, occurs after all the antidiuretic hormone (ADH) has leaked out of the damaged neurons. This third phase may not occur as severe pituitary damage must be present for a majority of vasopressin-secreting neurons to be destroyed [4,5]. Close monitoring of serial laboratory values is necessary to determine the patient’s clinical status as they proceed through the phases. 

## Conclusions

This report details both presentation and treatment of pituitary apoplexy. When diagnosis is suspected, the gold standard imaging of choice should be MRI, however, CT is usually done first because it is more efficient to obtain. Treatment includes serial monitoring of electrolytes and fluid balance. In addition, all patients should receive stress-dose corticosteroids whether or not they have symptoms of adrenal insufficiency. Surgery should be performed with hypothalamic involvement, deteriorating consciousness, and rapid, progressive visual disturbances. This case shows a prime example of identifying broad symptoms and timely treatment resulting in positive outcomes.

## References

[1] Ranabir S, Baruah MP (2011). Pituitary apoplexy. Indian J Endocrinol Metab.

[2] Shou XF, Wang YF, Li SQ, Wu JS, Zhao Y, Mao Y, Zhou LF (2009). Microsurgical treatment for typical pituitary apoplexy with 44 patients, according to two pathological stages. Minim Invasive Neurosurg.

[3] Veldhuis JD, Hammond JM (1980). Endocrine function after spontaneous infarction of the human pituitary: report, review, and reappraisal. Endocr Rev.

[4] Hoorn EJ, Zietse R (2010). Water balance disorders after neurosurgery: the triphasic response revisited. NDT Plus.

[5] Mayol Del Valle M, De Jesus O (2022). Pituitary Apoplexy. https://www.ncbi.nlm.nih.gov/books/NBK559222/.

